# Hidden Threats: The Unnoticed Epidemic System of Pine Wilt Disease Driven by Sexually Mature *Monochamus* Beetles and Asymptomatic Trees

**DOI:** 10.3390/biology14050485

**Published:** 2025-04-28

**Authors:** Kazuyoshi Futai, Hideaki Ishiguro

**Affiliations:** 1 Graduate School of Agriculture, Kyoto University, Kyoto 606-8502, Japan; 2 Ishiguro Tree Doctor Office, Suzuka 513-0012, Japan; info@ishiguro.cc

**Keywords:** pine wilt disease, asymptomatic infected tree, *Monochamus* beetle, *Bursaphelenchus xylophilus*, infection cycle, sexually mature beetle

## Abstract

Pine wilt disease, caused by the nematode *Bursaphelenchus xylophilus*, poses a significant threat to pine forests worldwide. Understanding the dynamics of its spread is crucial for effective disease management. In this study, we investigated the involvement of asymptomatic carrier trees in the expansion of pine wilt disease through a series of experiments. Cage-releasing experiments revealed that sexually immature Japanese pine sawyer beetles, *Monochamus alternatus*, feeding on healthy pine branches drops only a minimal number of nematodes (primary infection). However, sexually mature beetles, still harboring numerous nematodes, fly to weakened trees for breeding and extend their feeding activities to healthy pines around weakened trees, infecting them with nematodes and thus spreading the disease further. Inoculation experiments on field-planted black pine seedlings demonstrated that even a small number of nematodes can lead to a high occurrence of asymptomatic carrier trees. Our findings suggest that nematode infections transmitted by sexually mature *Monochamus* beetles significantly contribute to the expansion of pine wilt damage and play a crucial role in the persistence of asymptomatic carrier trees. This conclusion is based on cage-release experiments demonstrating nematode transmission by mature beetles and inoculation experiments highlighting the conditions leading to asymptomatic carrier trees.

## Simple Summary

Based on robust results from in-cage vector beetle release experiments and pine seedling inoculation trials, we demonstrate that sexually mature *Monochamus* beetles, attracted to withering trees for reproduction, can transmit pathogenic nematodes to surrounding healthy trees. This leads to the formation of new asymptomatic carrier trees, which in turn facilitates the spread of pine wilt disease and may nullify the effectiveness of current control efforts.

## 1. Introduction

Pine wilt disease (hereafter abbreviated as PWD), responsible for significant damage to pine forests throughout Japan except for Hokkaido, peaked around 1980, resulting in an annual loss of over 2 million cubic meters of pine trees nationwide. Following this peak, the disease appeared to gradually subside. However, this apparent decline can be attributed to the significant reduction in pine forests susceptible to the disease. Presently, pine wilt disease is spreading to the last remaining forests of Japanese red pine, *Pinus densiflora* Sieb. et Zucc. in cold high-altitude areas and high-latitude regions in Japan [1]. Additionally, due to the increased global movement of materials, this disease, which has devastated Japanese forests, spread to other East Asian countries [2,3] and reached Portugal in Europe by the end of the 20th century [4], later spreading to Spain [5] and Armenia [6].

**Figure 1:**
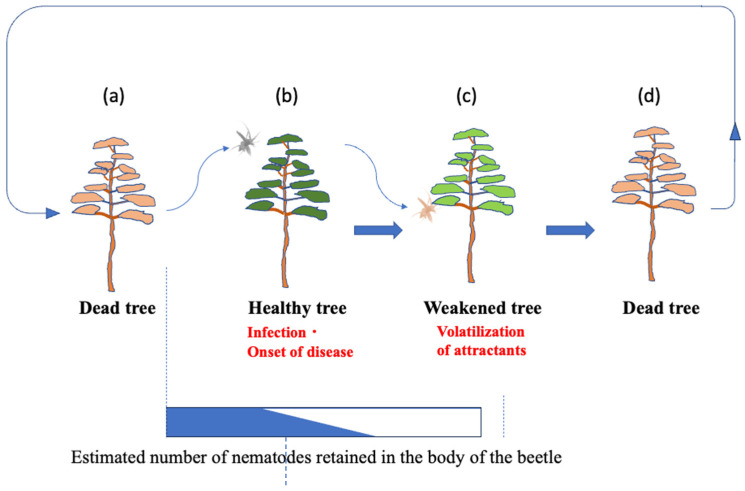
Established theory on the infection chain of PWD. The rectangular bar at the bottom of the figure shows the gradually decreasing percentage of nematodes (blue) retained in the body of the *Monochamus* beetle. Descriptions in red refer to the disease status of the host tree.

The established infection cycle of pine wilt disease (PWD) has been understood as follows: Pine sawyer beetles with immature reproductive organs emerge from pine trees that died due to PWD the previous year [7]. To mature their reproductive organs, beetles fly to healthy pines and vigorously feed on the bark of young branches, creating feeding scars. This period of sexual immaturity lasts about 5–15 days [8] for males and 16–30 days [9] for females, during which the feeding activity creates scars on the bark. Nematodes retained within the beetle’s body enter the pine tissues through these feeding scars, initiating infection (primary infection). As the host pine becomes diseased, it emits volatile compounds such as ethanol and terpenes, attracting sexually mature beetles that fly to weakened pines for mating and oviposition. Beetles overwinter as matured larvae within the dead pine wood, pupate the following spring, and emerge as adults carrying numerous nematodes from the dead wood. These adults then fly to healthy pines, perpetuating the infection cycle (Figure 1).

Based on this understanding of the infection cycle, two primary methods have been utilized to control this forest epidemic in Japan. One strategy entails the preventive application of insecticides on host pine trees to deter the vector, the Japanese pine sawyer beetle, *Monochamus alternatus* Hope (Japanese pine sawyer beetle, hereafter referred to as *Monochamus* beetle, or beetle), from feeding on young branches. This prevents the pathogen, the pine wood nematode *Bursaphelenchus xylophilus* (Steiner & Buhrer) Nickle (hereafter abbreviated as PWN or Bx nematode), harbored within the beetle’s body from invading the tree tissues via the beetle’s feeding scars. The other method involves the eradication of dead trees, where both the nematodes and beetle larvae breed, through felling and removing by burning or fumigation. Despite the understanding that thorough implementation of these methods should prevent damage, in reality, even after felling and removing all dead trees in the forest in early spring, a few wilting trees due to PWD occur surrounding the stumps of removed dead trees in early summer [10]. To understand the reasons behind these puzzling patterns of damage occurrence, one of the authors conducted a thorough investigation over four years focusing on a total of 72 Korean pine trees (*Pinus koraiensis* Sieb. et Zucc.), each approximately 40 years old and planted in an arboretum [11]. This investigation involved tracking the locations of dead trees while continuously monitoring resin exudation [12], a physiological indicator of pine health, in all trees starting from the year when two dead trees first appeared due to pine wilt. It is important to note that the damage in this forest continued to increase, even though all dead wood had been completely removed by early summer, prior to the appearance of the beetles. This removal was intended to prevent damage from occurring and spreading further throughout the forest. As a key to solving this mystery, we incorporated the concept of “**asymptomatic infected trees**”. These are trees that become infected with nematodes in a given year and experience a decline in resin exudation but survive without visible symptoms until the following year or later. As a result, they are overlooked during the eradication of dead trees by early summer and later develop visible symptoms, contributing to disease progression [11].

To reassess existing theories on the PWD infection cycle and clarify the role of asymptomatic carrier trees in disease spread, it is essential to quantify the number of nematodes carried by beetles arriving at these trees for reproduction. Additionally, a detailed investigation into the relationship between their feeding behavior during reproduction and nematode transmission to healthy trees is required. Therefore, in this study, we initially observed the behavior of beetles towards both asymptomatic carrier trees and healthy trees nearby and assessed nematode infection in the healthy trees through cage release experiments.

Furthermore, to elucidate the conditions under which asymptomatic carrier trees arise as breeding targets for beetles, we inoculated 3- or 5-year-old Japanese black pines, *Pinus thunbergii* Parl, in nurseries, and potted 3-year-old Japanese black pines with a small number of nematodes in early and late summer. We then observed the progression of disease until the following summer. Additionally, we regularly harvested inoculated seedlings and isolated nematodes from various parts of the seedlings to determine their distribution within the host plant and its association with their physiological and external symptoms.

## 2. Materials and Methods

The following in-cage experiments and the field inoculation experiments were implemented in Suzuka City, Mie Prefecture (34°52′ N, 136°35′ E)

**1. In-Cage Insect release experiments: Investigation of Beetle Reproductive Behavior and Transition of Retained Nematodes to Surrounding Healthy Trees**

As some of the results of the in-cage experiments have already been published in another Japanese journal [13], a summary of the methods and results is given here to aid understanding of the overall purpose of this experiment. For statistical details of the results of the in-cage experiments, see the original paper [13].

**Figure 2:**
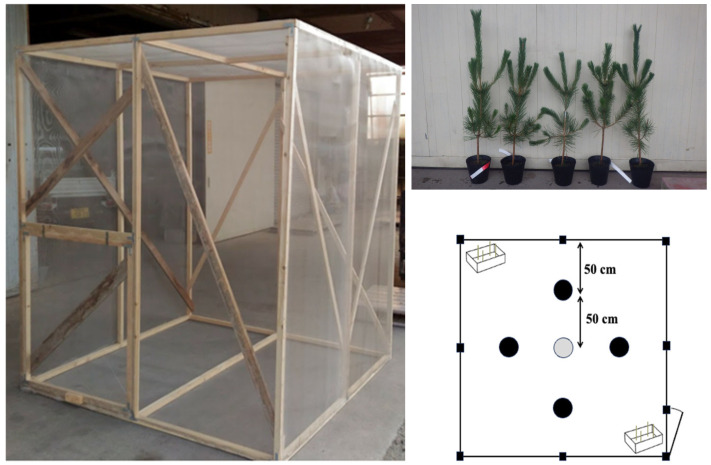
Mesh cage used for the *Monochamus* beetle release experiments (left). Five potted pine seedlings were used in each experiment: the seedling placed at the center was an artificially latently infected tree inoculated with nematodes (top right). The arrangement of the seedlings within the cage is shown in the schematic diagram (bottom right). An artificially made latent carrier seedling was pre-inoculated with 5000 *Bx* nematodes, and its cessation of resin exudation was confirmed 2 days prior to each experiment, then placed in the center. Insect release boxes were placed in two corners of the cage. In each, three male or three female sawyer beetles were placed. (Modified and reproduced from Ishiguro and Futai [13]).

The experiments were conducted in a mesh cage measuring 2 m in height, width, and depth. About a month before the cage release experiments, one externally healthy three-year-old black pine seedling was designated as the oviposition target tree —essentially an artificially created asymptomatic carrier tree. To induce disease onset, indicated by the cessation of resin exudation, the seedling was pre-inoculated with 5000 pathogenic nematodes. This seedling was placed at the center of the cage, surrounded by one healthy black pine seedling in each of the four directions. Positioned in two corners of the cage were polystyrene foam boxes (dimensions: height × width × depth = 15 × 18 × 20 cm). Within each box, four wooden sticks, each 20 cm in length, were erected to facilitate natural takeoff for the beetles (Figure 2).

Pine logs that had been killed due to pine wilt in Ueno Forest Park, 43 km away, were transported and stocked in a 1.5 × 2 × 2 m (H × W × D) netted cage. Beetles emerging from the logs were collected daily, and their size, sex, and the date of collection were recorded and each beetle was kept in a plastic container (1–4 L) with a small hole for ventilation until each in-cage-release experiment. A short Japanese black pine twig was used as food and replaced with a fresh one every two to three days.

Three unmated females were released into one box, and three unmated males were released into the other. After 72 h, the number of feeding scars on each of the five black pine seedlings and the number of nematodes isolated from each feeding scar were investigated. Following previous reports [8,9], mature and immature beetles were discriminated based on the number of days after emergence. The experiments were repeated nine times for sexually immature beetles (males aged 5–10 days, females aged 5–10 days) and sexually mature beetles (males aged 14–31 days, females aged 19–30 days), respectively. Furthermore, following each release test, each tested beetle was individually dissected, and nematodes remaining in the beetle body were isolated using the Baermann funnel method and counted.

**2. Examination of the Conditions under which Asymptomatic Carrier Trees Occur: Experimental Inoculation of Pine Seedlings Planted in the Field with a Small Number of Nematodesl**

Some trials of the cage experiments showed that the number of nematodes invading through a single *Monochamus* beetle feeding scar was generally low. Therefore, in the second series of inoculation experiments, we set the inoculation dose to a lower level of 50 nematodes per site (50 ± 8.3). Two experiments were conducted: The first experiment aimed to determine how host pines develop disease when infected with a small number of nematodes in different seasons and to examine the distribution and quantity of nematodes in trees that survive as asymptomatic carrier trees. The second experiment investigated the relationship between the number of beetle feeding scars and the mortality rate of pine saplings.

### 2.1. Differences in Disease Development and Nematode Distribution in the Host Tree According to Infection Time

Previous inoculation experiments have experienced that late inoculation often results in delayed onset of symptoms until the following year [14]. Additionally, it is known that physiological changes in pines seem to occur in late stages of the pine wilt season (late August to September), affecting the infection of pathogenic nematodes [15]. To confirm this, 50 nematodes were inoculated on the one-year-old branches of 80 seedlings of three-year-old black pine in the early pine wilt season (1 July 2018) and on 65 seedlings in the late pine wilt season (25 August 2018). Subsequently, 20 seedlings designated from each inoculation timing group were separated from others for periodical observation for disease progression, recording resin exudation status as physiological symptoms and changes in external symptoms through photography from 29 June just prior to nematode inoculation to 30 July of the following year for the early inoculation group and, similarly, from 22 August to 30 July of the following year for the late inoculation group. Although designated control plots were not established due to operational constraints, dozens of untreated healthy seedlings growing in the same nursery were continuously monitored throughout the experiment. These untreated seedlings served as an informal control group, allowing comparison of symptoms with those of the nematode-inoculated seedlings.

Furthermore, to determine in which part, for how long, and how many nematodes survived, a total of 11 times after inoculation on 1 July, 2018 until April of the following year for the early inoculation group and 7 times after inoculation on 25 August, 2018 until April of the following year for the late inoculation group, five randomly selected inoculated seedlings from each inoculation timing group were collected periodically, using a random number table. From each of the collected seedlings, five tissue samples were taken: (1) the inoculation site, (2) another branch of the same whorl of the inoculated branch, (3) the upper part, (4) the lower part of the main stem, and (5) the roots. Each sample was shredded and soaked in a small Baermann apparatus for 24 h, and the isolated nematodes were counted under a stereomicroscope. After nematode isolation, each pine tissue was air-dried, weighed, and the number of nematodes was evaluated per gram of dry weight.

### 2.2. Relationship Between the Number of Beetle Feeding Scars and Mortality Rate of Pine Saplings: Effect of Multipoint Infection

When beetles feed on healthy pines, they create feeding scars in multiple places while walking on branches. In the cage-release experiments, it was observed that, on average, each beetle left about four feeding marks on the seedlings during a three-day observation period. Even with a low number of nematodes per site, an increase in the number of feeding sites and total nematodes infecting a single seedling may increase damage to the host pine and raise the probability of death. To investigate this, 50 saplings of five-year old black pine planted in the field were inoculated with 50 nematodes per site, varying the number of inoculated sites (number of nematodes per sapling) from 1 site (50) to 2 sites (100), 3 sites (150), 5 sites (250), and 7 sites (350), on 21 July 2017. Ten saplings were used for each number of inoculation sites (number of inoculated nematodes). After inoculation, resin exudation (symptom) of each sapling was observed regularly.

In experiments 2.1 and 2.2, the inoculum used for inoculation was the highly pathogenic *B. xylophilus*, Ka-4 strain cultured on *Botrytis cinerea* (grey mold) colonies. The pathogenicity of the nematodes used for inoculation was confirmed before each experiment by inoculating them into five healthy black pine trees (at a rate of 1000 nematodes per seedling and 4500 nematodes per sapling), resulting in nearly complete tree mortality.

In all experiments, the progression of symptoms was assessed based on visual changes observed and judged from photo recordings, while physiological symptoms were assessed based on the amount of resin exuding from pinholes made in the main stem of seedlings two hours after pinning, which is a modification of Oda’s method [12] for small seedlings.

## 3. Results

### 3.1. In-Cage Insect-Release Experiments

#### 3.1.1. Number of Nematodes Invading Feeding Scars Left on Healthy Trees Surrounding Breeding Target Trees

**Figure 3:**
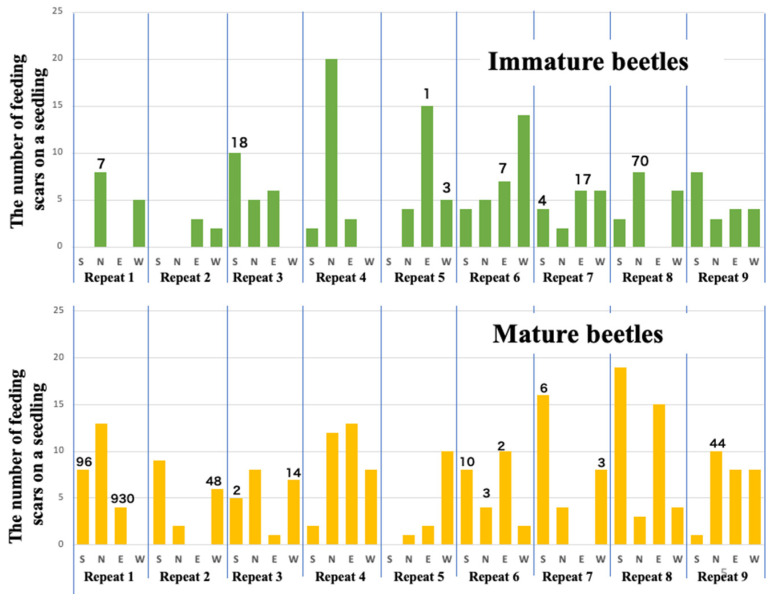
The number of feeding scars and infecting nematodes per seedling. Top: immature beetles, bottom: mature beetles. The numbers above some of the bars indicate the total number of nematodes that invaded from the scars on each seedling. The abbreviations N, S, E, W on the *x*-axis indicate directional orientation of each seedling.

In experiments with sexually immature beetles, no significant difference was observed in the number of feeding scars left on black pine seedlings between the healthy trees placed around and the weakened trees placed in the center. Among the 36 healthy trees tested, feeding scars were observed on 28 trees (77.8%), totaling 224 scars. However, examination of 145 feeding scars revealed only 127 nematodes detected from a total of 17 scars on eight healthy trees combined (Figure 3 top). Despite the mean number of nematodes retained by the 54 sexually immature beetles used in this experiment being low, at 686 with a standard deviation of 2248, it is noteworthy that two individuals retained over 10,000 nematodes each and three beetles retained between 1000 and 10,000 nematodes. This suggests that nematodes retained by beetles during their sexually immature period very rarely fall into feeding scars, and the number of nematodes invading pine tissues is limited to a very small extent (Table 1).

However, sexually mature beetles exhibited a notable difference in behavior compared to sexually immature beetles. They left significantly more feeding scars on the asymptomatic carrier trees placed in the center of the cage than on the healthy trees placed around them (Tukey’s multiple comparison test, *p* < 0.05). However, they also left feeding scars evenly on the healthy trees placed in all four directions. In other words, sexually mature beetles were observed not only to be attracted to weakened (asymptomatic carrier) trees for reproductive activities, where they mated and laid eggs, but also to continue vigorous feeding activity during this reproductive period moving between these oviposition target seedlings and surrounding healthy seedlings [13].

Feeding scars, which are traces of their feeding activities, were observed on 32 (89%) of 36 healthy trees tested in the nine repeat trials. The total number of feeding scars on these 32 trees was 231, which was nearly the same as that observed in the case of immature individuals (*t*-test, df = 69, *p* = 0.148). Examination of nematodes invading 196 of these feeding scars revealed nematodes detected from 20 scars (Figure 3 bottom). The average number of nematode infections per seedling was approximately 35, which was ten times higher than the average infection rate of approximately 3.5 nematodes per seedling by immature beetles. However, the standard deviation was large in this case, so it cannot be immediately concluded that mature beetles infect more nematodes (*t*-test, df = 35, *p* = 0.240).

As shown above, *t*-tests were conducted to compare the mean number of feeding scars per seedling and the mean number of nematodes infected per seedling between immature and mature beetles. However, no significant differences were detected (*p* > 0.05) because large variances and overlapping distributions were observed in the data. Therefore, we limited our analysis to summary and basic significance testing, which we judged sufficient for this comparison.

Due to the experimental design—where feeding behavior was measured as the cumulative outcome of three females and three males on four healthy trees (and one asymptomatic carrier tree) per replicate—it was not possible to calculate individual-level variation in beetle behavior.

#### 3.1.2. The Number of Nematodes Remaining in the Beetle Bodies After the Release Test

Furthermore, when comparing the number of nematodes remaining in the beetle bodies after the release test between 53 mature individuals and 54 immature individuals, it was found that mature individuals retained approximately twice as many nematodes (1526.0 ± 3918.6 *) as immature individuals (686.3 ± 2248.0 *). However, due to the large standard deviation, the difference was not significant (*t*-test, df = 102, *p* = 0.211). * denotes average ± standard deviation.

Nevertheless, from this result, it can be concluded that, at least, contrary to the previously believed notion, the nematodes retained by sexually mature individuals are not significantly fewer than those retained by immature individuals.

Furthermore, the cage-release tests revealed that the number of nematodes infecting from one feeding scar of the beetle is often below several tens, indicating that the infection of thousands to tens of thousands of nematodes from a single wound, as has been commonly used in many inoculation experiments, very rarely occurs.

### 3.2. Experimental Inoculation of Pine Seedlings Planted in the Field with a Small Number of Nematodes

#### 3.2.1. Differences in Disease Development and Nematode Distribution in the Host Tree According to Infection Time

When a small number (50 nematodes per tree) of *Bx* nematodes were inoculated in early pine wilt season (1 July 2018), resin abnormality occurred by the fourth week, reaching 80% of the inoculated seedlings by the eighth week (22 August). In addition, external symptoms appeared in 35% of the inoculated seedlings in the tenth week after inoculation, presenting as whole or partial discoloration, and this proportion rose to 60%, of which 35% were totally dead and 25% partially dead, by the 13th week. However, the remaining 40% seedlings remained asymptomatic until the end of July the following year.

**Figure 4:**
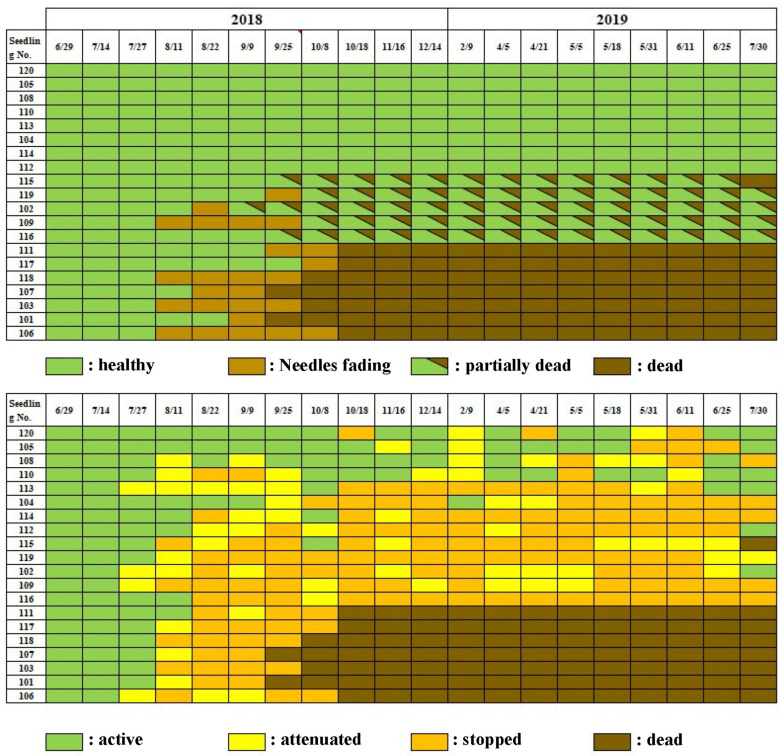
Progress of external disease symptoms (**top**) and resin exudation decline (**bottom**) in black pine seedlings inoculated with small number of nematodes early in the pine wilt season.

Among the seedlings that exhibited resin abnormality, half withered by the beginning of the following season, while the remaining half survived without withering until the end of the observation (30 July 2019) (Figure 4).

**Figure 5:**
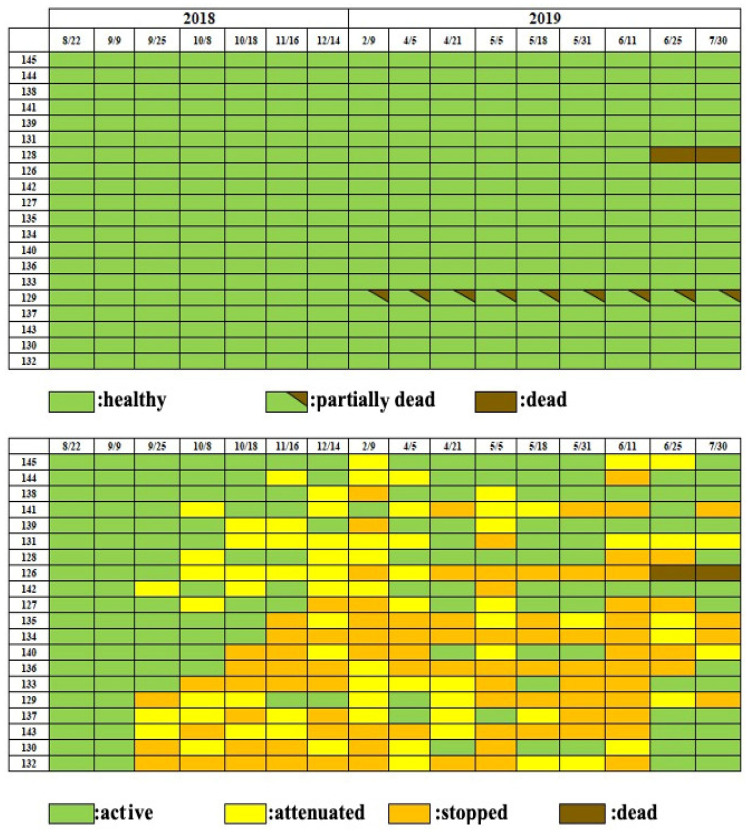
Progress of external disease symptoms (**top**) and resin exudation decline (**bottom**) in black pine seedlings inoculated late in the pine wilt season.

On the other hand, when a small number of *Bx* nematodes (50 individuals per seedling) were inoculated on 25 August 2018, late in the season, seedlings showing resin abnormality appeared four weeks after inoculation. By the eighth week (15 October), this proportion reached 50% of the inoculated individuals. However, external symptoms such as discoloration or fading did not appear at all until 9 February of the following year, when discoloration appeared on one branch of one seedling, while the other seedlings maintained a healthy appearance until the end of the experiment, except for two. Among the two showing abnormalities, one was the seedling with branch discoloration mentioned above, and the other withered in the tenth month after inoculation. Thus, when the inoculation timing was late, the decrease in resin exudation, an internal symptom, was similar to that of early inoculation timing. However, the leaf color of most seedlings remained healthy until the next pine wilt season (Figure 5).

**Figure 6:**
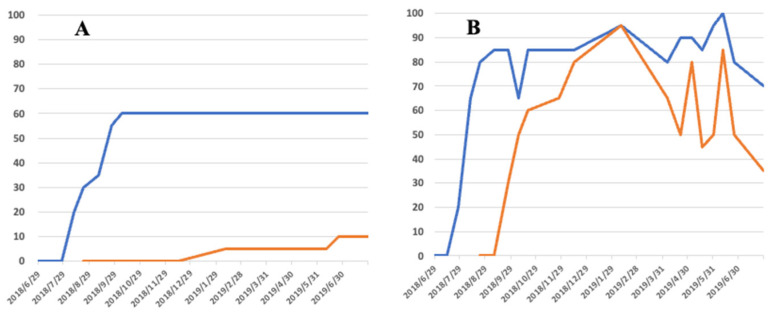
Comparison of disease progression between early and late inoculation groups. (**A**) Percentage of dead seedlings over time. (**B**) Percentage of seedlings showing reduced or stopped resin secretion over time. 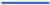: Early inoculation group; 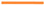: Late inoculation group.

The chart formats used in Figure 4 and Figure 5 were designed to visualize symptom progression and resin response in individual seedlings over time. In addition, Figure 6 summarizes group-level trends in disease progression as a time-series line graph.

**Figure 7:**
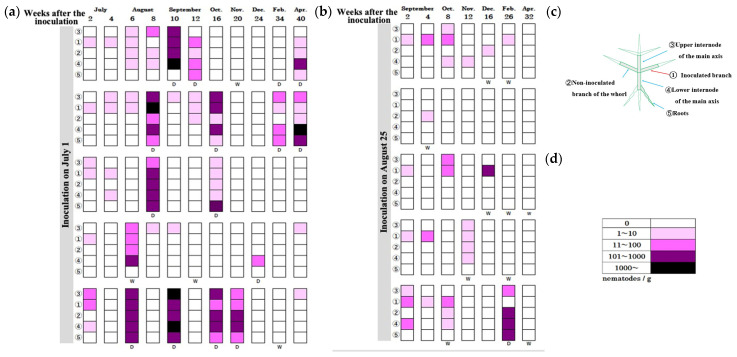
The distribution trend of the inoculated nematodes within the host seedling when inoculated in the early season, 1 July (**a**) and the late season, 25 August (**b**). As illustrated in the figure (**c**), tissue samples were taken from five points of the seedlings, including ① the inoculation site, ② other branches at the same branch level as the inoculated branch, ③ the upper part of the main stem, ④ the lower part of the main stem, and ⑤ the roots. Nematode density per gram of dried tissue was expressed as five levels of color shade (**d**).

As described above, when a small number (50 individuals) of nematodes were inoculated in early pine wilting season (1 July) compared to late inoculation (25 August), physiological abnormalities progressed similarly, but the expression of external symptoms differed significantly. How did the distribution trend of the inoculated nematodes within the host tree differ between the two inoculation times? In the case of inoculation in July, nematodes not only spread within the seedlings three to four weeks after inoculation, but instances of successful proliferation were also observed, leading to an increase in weakened (denoted as W in the figure) and dead seedlings (denoted as D) accordingly (Figure 7a).

On the other hand, when inoculated late, it appears that the proliferation of nematodes within the seedlings is suppressed, perhaps due to changes in the physiological status of the seedlings in response to seasonal climatic changes (Figure 7b). In both cases, nematode proliferation within the seedlings is reduced after November, possibly due to low temperatures, and the number of seedlings from which nematodes cannot be isolated increases. However, this does not mean that nematodes completely disappear from the seedlings. Some seedlings with a healthy appearance still yielded a small number of nematodes even in the following February and April.

#### 3.2.2. Relationship Between the Number of Beetle Feeding Scars and Mortality Rate of Pine Saplings: Effect of Multipoint Infection

**Figure 8:**
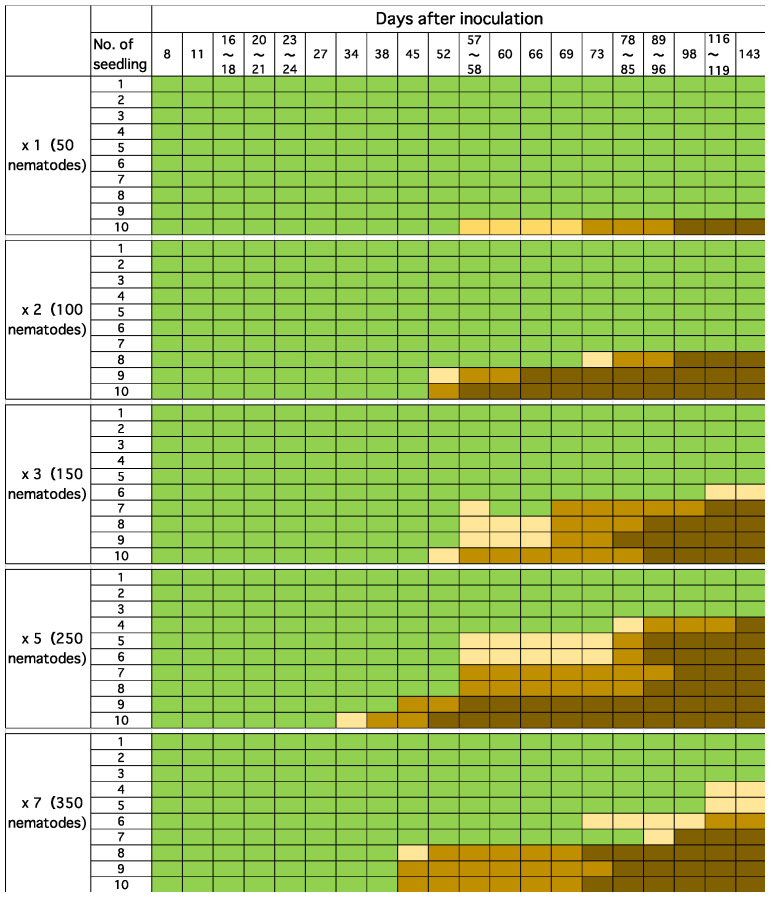
The relationship between the number of inoculation points (inoculated nematodes) and the mortality rate. Green, beige, light brown, and dark brown represent healthy, resin decline, resin stop, and withering, respectively.

**Figure 9:**
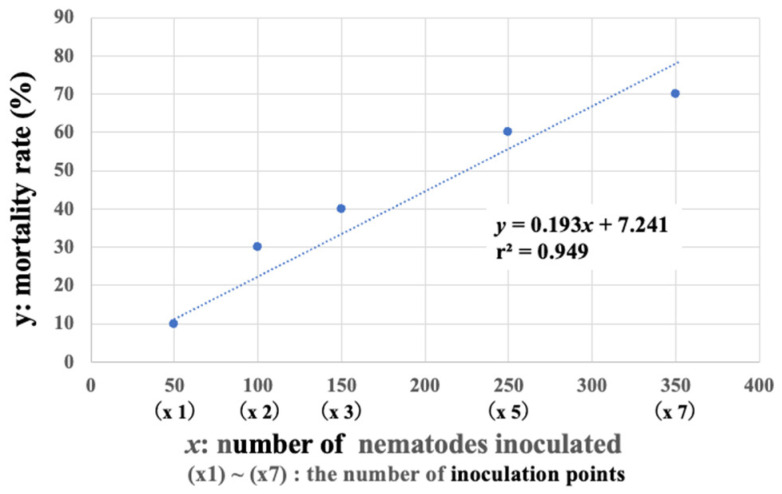
Regression analysis of the relationship between the number of inoculation points (nematodes inoculated) and the mortality rate.

As the number of inoculation points per sapling increased, individuals exhibiting abnormal resin exudation appeared earlier and a higher proportion of individuals eventually died (Figure 8). When examining the relationship between the number of inoculation points and the mortality rate at five months (143 days) post-inoculation, a strong positive correlation (r^2^ = 0.949) was observed (Figure 9). This experimental result suggests that even if the number of nematodes per feeding scar is small, pine saplings become more susceptible to wilting when multiple feeding scars occur.

## 4. Discussion

Our study challenges conventional assumptions about the infection cycle of pine wilt disease (PWD). In the cage-release experiment, sexually mature beetles (males aged 14–31 days, females aged 19–30 days) exhibited distinct feeding behavior compared to sexually immature beetles (males aged 5–10 days, females aged 5–10 days). They created significantly more feeding scars on asymptomatic carrier trees placed at the center of the cage than on healthy trees positioned around them (Tukey’s multiple comparison test, *p* < 0.05). However, they also left feeding scars evenly across all healthy trees. This suggests that, contrary to previous understanding, sexually mature beetles are not exclusively attracted to weakened or dead trees for reproduction. Instead, they continue vigorous feeding during their reproductive period, moving between oviposition sites and surrounding healthy seedlings [13].

Previously, it was believed that sexually immature *Monochamus* beetles transmitted most of the nematodes they carried through feeding scars on healthy pines, causing primary infections (Figure 1). However, our cage-release experiments revealed that nematode transmission by immature beetles was minimal; very few nematodes entered feeding scars, and only a limited number successfully invaded pine tissues (Table 1). This aligns with earlier reports showing that pine trees fed on by immature beetles within the first 10 days post-emergence exhibited no mortality, whereas trees fed on by sexually mature beetles 2 to 7 weeks post-emergence showed high mortality [16]. Additionally, previous studies indicate that nematodes use intestinal storage lipid reserves as an internal clock, exiting the beetle only after a certain amount of lipid is consumed [17].

On the other hand, it has long been assumed that sexually mature beetles arriving at weakened trees for reproduction carry few nematodes, as most nematodes were thought to have already left the beetle’s body during its earlier feeding activity on healthy pines. However, our experiments challenge this assumption. In the cage-release study, sexually mature beetles were individually maintained on young pine branches before reaching sexual maturity (~10 days for males, ~20 days for females). Even after feeding on pine seedlings for three days during the experiment, dissection revealed that males still retained an average of 850 nematodes and females an average of 1900. This suggests that sexually mature beetles actually harbor far more nematodes than previously believed when they arrive at weakened trees for reproduction. The results of our cage-release experiment, as shown in Table 1, suggest that sexually mature beetles transmit nematodes to healthy trees surrounding weakened trees (latent carrier trees) as sexually mature beetles are attracted to the weakened trees for reproductive activity.

Our findings are supported by studies on the timing of nematode transmission. Togashi [18] and Naves et al. [19] found that peak nematode transmission occurred 20–35 days and 15–42 days after beetle emergence, respectively, coinciding with sexual maturation and attraction to declining trees. Once these beetles begin reproduction, they continue feeding on both the weakened trees they inhabit and nearby healthy trees, thereby establishing new infection routes. However, the role of sexually mature beetles in transmitting nematodes to healthy trees has been largely overlooked.

In our experiments, sexually mature beetles frequently fed on healthy trees surrounding weakened ones (32 out of 36 healthy trees tested), creating 231 feeding scars—similar in number to those made by immature beetles. When we examined 196 of these scars, 20 contained nematodes, with an average of 35 nematodes per seedling. Importantly, even a small number of invading nematodes can have significant consequences. As demonstrated in Experiment 2b, an increase in feeding scars, rather than the size of individual scars, leads to greater nematode entry, accelerating disease progression. Interestingly, previous research found no correlation between feeding scar size and the number of transmitted nematodes [20], reinforcing the idea that total feeding site number is the primary determinant of transmission efficiency.

The results of our nematode inoculation experiment further support the role of latent infections. In the early-season inoculation experiment (1 July 2018), where a small number (50 nematodes) was introduced, disease progression was slow. Among 20 monitored black pine seedlings, 40% were completely dead, and 20% were partially dead by July of the following year (Figure 4). Partial wilting is often overlooked in control efforts, yet studies on *Pinus koraiensis* forests revealed that even after wilted branches were removed, 7 out of 13 trees eventually died [11]. These findings highlight the importance of monitoring not only symptomatic trees but also asymptomatic carrier trees and those with partial wilting.

Notably, late-season inoculations resulted in fewer cases of severe wilting, with most trees remaining asymptomatic until the following year or later. This suggests that certain infection conditions contribute to the formation of latent carrier trees. One key factor is the low number of infecting nematodes. Some infected trees survive without visible symptoms yet harbor small nematode populations internally. Increased temperatures and drought conditions [21] can weaken host resistance, triggering nematode proliferation, disease progression, and the eventual creation of breeding sites for sawyer beetles. Another factor is lower temperatures during late-summer or early-autumn infection, which may suppress nematode activity and reproduction and thereby enhance host resistance keeping in healthy looking trees the following year. In essence conditions that slow disease development contribute to asymptomatic infections. Even when trees at the same site are infected simultaneously, individual variations in these factors can determine whether a tree dies or becomes a latent carrier [22].

The ease of nematode invasion from beetle feeding scars is a critical factor in host susceptibility. This property varies by pine species and seasonal conditions [15]. In susceptible black pines, only about 10% of nematodes at feeding scars successfully invade host tissues [23]. Since *B. xylophilus* reproduces sexually, very low infection densities could reduce mating opportunities, leading to population collapse. However, nematode sex pheromones facilitate encounters between males and females [24], enhancing population persistence. If reproduction fails, the nematodes disappear and the tree survives. If successful, the nematode population remains at low density until host resistance declines, triggering rapid proliferation and wilting. This was evident in our experiments, where nematodes were recovered from symptom-free seedlings the following April.

As mentioned above, however, it is often difficult to detect low density of nematodes in plant tissue by the Baermann funnel method. Molecular tools such as PCR-based methods [25,26] and the LAMP method [27] that are more sensitive and applicable to field samples would be needed to improve nematode detection sensitivity in future studies of asymptomatic carriers. These molecular approaches offer the advantage of high-throughput screening without requiring taxonomic expertise, making them suitable for rapid and large-scale diagnostics. Nevertheless, due to their relatively high cost, such methods are not practical for long-term monitoring studies like ours, which require repeated sampling of a large number of trees over time.

Long-term latent infections have also been observed in other pine species. Bergdahl and Halik [28] found that in Vermont, USA, despite inoculating 30,000 nematodes, many *Pinus sylvestris* (Scots pine) trees survived for 7–11 years as asymptomatic carriers, with nematodes persisting for 2–11 years. The cooler climate and higher resistance of *P. sylvestris* compared to *P. thunbergii* may have contributed to this prolonged latent phase [29], but the study nonetheless illustrates how nematodes can persist long term in asymptomatic carriers.

**Figure 10:**
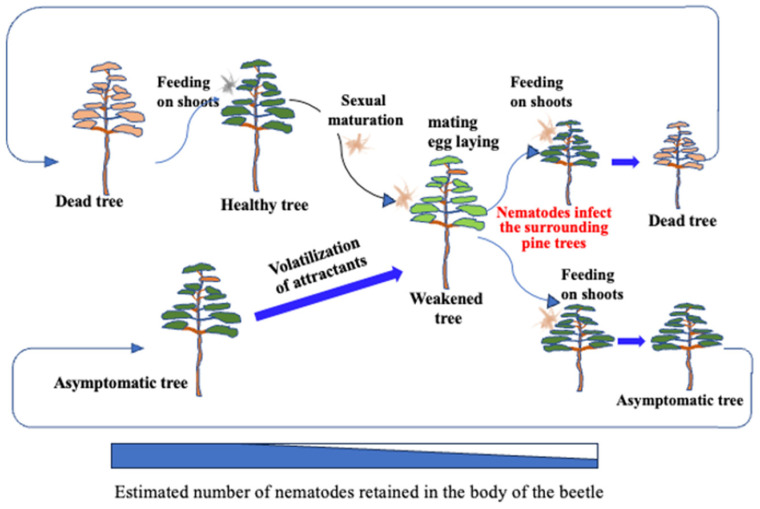
New understanding of the PWD infection chain. Immature beetles moving among the pines are shown in gray, while mature beetles are shown in light brown. The explanation shown in red text is most important phase of disease expansion.

After dead trees are removed from a forest, sawyer beetles may migrate and feed on remaining healthy trees. However, these trees take time to develop disease symptoms and reduced resin exudation, which makes them suitable for egg-laying. Research suggests that a one-month gap exists between beetle emergence and the onset of resin-abnormal trees [30]. Because *Monochamus* beetles lack suitable oviposition sites for ~20 days (males) and ~10 days (females) after emergence, latent infections from previous years provide critical breeding sites, ensuring population continuity. This means that mature beetles contribute significantly to outdoor nematode transmission by introducing small numbers of nematodes into multiple feeding sites on healthy trees surrounding weakened hosts (Figure 10). This overlooked process underscores the importance of asymptomatic carriers in sustaining PWD, nullifying control efforts and facilitating disease spread.

Recent advancements in PWD detection methods have improved monitoring [31]. Aerial detection using remote sensing and UAVs (Unmanned Aerial Vehicles) identifies symptomatic trees at a large scale but remains ineffective for detecting asymptomatic carriers [32,33,34,35]. Direct detection methods, such as volatile compound analysis [36,37], DNA-based techniques [25,38,39], and pH measurement of wood samples [40], offer high accuracy but are costly and impractical for large-scale surveys.

Traditional resin exudation measurement remains the most practical approach for detecting infected trees in the field [12,41].

To explore the progression of nematode distribution within the seedlings, we employed a time-consuming method, but many seedlings with no visible symptoms yielded no nematodes upon isolation. This difficulty arises from the methodological constraints of the Baermann funnel technique, which makes it challenging to completely isolate low-density nematodes within plant tissues. Therefore, around November, when the number of isolates decreased, we collected samples from visually healthy seedlings. We divided these samples into two, with one part immediately undergoing nematode isolation and the other left at room temperature for five days to promote nematode proliferation before attempting isolation. As a result, among five seedlings where nematodes were not initially detected, nematodes were successfully isolated from three seedlings after the second attempt.

## 5. Conclusions

Finally, our study highlights the importance of revisiting traditional views on the pine wilt infection cycle. Asymptomatic carrier trees play a critical role as hidden drivers of pine wilt disease spread—not by directly harboring high nematode loads but by attracting sexually mature *Monochamus* beetles that subsequently disperse and infect pathogenic nematodes to surrounding healthy trees. Recognizing this behavioral and ecological role of mature beetles is essential for effective PWD management.

## Figures and Tables

**Table 1:** Number of Feeding Scars and Nematodes per Surrounding Healthy Seedling Examined.

Number of Feeding Scars per Seedling Examined		Number of Nematodes Recovered per Seedling Examined	
Matured beetle	Immature beetle	Matured beetle	Immature beetle
**5.44** **± 4.05**	**4.02** **± 3.20**	**34.56** **± 155.15**	**3.53** **± 12.18**

## Data Availability

The data published in this study will be shared by the co-authors and are available upon request from the corresponding author.

## Abbreviations

The following abbreviations are used in this manuscript: **PWD**, pine wilt disease; **UAV**, Unmanned Aerial Vehicle.
